# IL-15 deficiency promotes tumor growth in tax transgenic mice

**DOI:** 10.1186/1742-4690-8-S1-A24

**Published:** 2011-06-06

**Authors:** Daniel Rauch, Sirosh Bokhari, John Harding, Lee Ratner

**Affiliations:** 1 Division of Molecular Oncology, St Louis, MO, 63110, USA

## 

IL-15 is an NFkB activated cytokine with structural similarity to IL-2 that has been implicated as a promoter of ATLL. Proposed mechanisms include; i) establishment of an autocrine loop in tumor cells that express both IL-15 and the IL-15 receptor, ii) IL-15 mediated protection from apoptosis, and iii) IL-15 mediated immune regulation that promotes tumor growth. IL-15 blockade is being considered as a therapeutic approach to HTLV-1 malignancies. To examine the effects of IL-15 deficiency on HTLV-1 malignancy in vivo, we developed IL-15-/- TAX-LUC mice in which firefly luciferase under the HTLV-1 LTR serves as a reporter of Tax expression driven by the granzyme B promoter. Unexpectedly, the absence of IL-15 resulted in a significantly enhanced tumor phenotype in TAX-LUC mice which developed markedly larger, more numerous, and more aggressive tumors. IL-15 deficiency also resulted in severe osteolytic disease, platelet and bone marrow abnormalities. Administration of soluble IL-15 slowed tumor growth in vivo. Characteristics of cell lines derived from IL-15-/- Tax tumors were indistinguishable from tumor cells derived from IL-15+/+ Tax tumors. RNA harvested from malignant and infiltrating cells within tumors indicated that tumor immunity is significantly affected by IL-15 loss. NK and gamma-delta T cells are diminished in tumor infiltrates in the absence of IL-15 and the malignant cells express elevated levels of IL-1alpha. These findings reveal a profound of effect of tumor immunity in TAX malignancies, implicate IL-1alpha as an important factor in the immune response to ATLL, and caution against IL-15 blockade as an ATLL therapy.

